# Supplementary material for: Individual and spatial heterogeneity of praziquantel efficacy against Schistosoma mansoni within the context of repeated mass drug administration
Source: Parasit Vectors. 2026 Jun 16;19:279. doi: 10.1186/s13071-026-07497-9 (PMC13343640; doi:10.1186/s13071-026-07497-9)
Supplement: Supplementary file 1 [file 13071_2026_7497_MOESM1_ESM.pdf]

Individual and spatial heterogeneity of praziquantel efficacy against *Schistosoma mansoni* within the context of repeated mass drug administration

Melissa A. Iacovidou[1], Fabian Reitzug[1], Sophie Winter[1], Annet Enzaru[2], Emily Asiimwe[2], Juliet Nambatya[3], Aisha Nakato[2], Moses Semakula[2], Betty Nabatte[2], Narcis B. Kabatereine[2], Goylette F. Chami[1*]

[1]Nuffield Department of Population Health, Big Data Institute, University of Oxford, Oxford, UK.
[2]Division of Vector Borne and Neglected Tropical Diseases, Uganda Ministry of Health, Kampala, Uganda.
[3]Buliisa District Local Government, Buliisa, Uganda.

*Corresponding author. E-mail: goylette.chami@ndph.ox.ac.uk

Supplementary information

Supplementary methods

Kato–Katz microscopy

Each team comprised one trained auxiliary worker and two technicians with many years of experience in Kato–Katz (KK) procedures. Trained auxiliary workers prepared the stool samples and slides for reading as follows:

1. Two glass slides were placed next to each other and labelled with the sample number plus a prefix A or B, and a clean plastic template was placed on top of each.
2. Stool was mixed using a clean spatula, and a small amount of stool was placed on a clean sieve.
3. Using the spatula, the sample was sieved through, ensuring that enough sample was available.
4. After cleaning the spatula, the faecal material was scraped from the underside of the sieve.
5. The hole in the templates was filled with the sample, making sure to avoid air bubbles and levelling the faces off to remove any excess. The same amount of stool was used to fill both slides. If the amount of stool was insufficient, one slide was prepared, and the participant was asked to bring another stool sample the following day.

6. Templates were carefully lifted off and placed in a bucket of water mixed with concentrated detergent to be reused later.
7. One piece of cellophane (previously soaked overnight in malachite green or methylene blue working solution) was placed over the faecal specimen.
8. The slides were inverted onto a flat surface covered with a clean newspaper and pressed firmly (starting from the sides of the slides and moving inwards in case the sample had not spread properly). The slides were left inverted for five minutes to ensure that any excess working solution was absorbed by the newspaper. Ideally, the newspaper should be legible through the stool smear.
9. The inverted slides were removed and sent for examination in the order of preparation.

The slides were read by the technicians approximately 24 hours after preparation for the examination of *Schistosoma* ova. The two technicians in each team were assigned either A- or B-labelled slides. When all slide readings were completed, the forms were findings were recorded on are reviewed for discrepancies. In the case of large discrepancies (one slide had a count 3 to 4 times the count of the other), the slides were identified and reread the following day. From each village, 10% of participants were randomly selected to have their slides reread for quality control in Kampala.

POC-CCA testing

Point-of-care circulating cathodic antigen (POC-CCA) tests (supplier: ICT International; product number: SCH25; lot number: 210811080 in 2022 and 221117133 in 2023) were also used to determine infection status. Technicians prepared the test cassettes by transferring two drops of urine to the circular well of the cassette from a well-mixed urine sample provided by the participants. Results were read approximately 20 minutes after preparation. A positive test was recorded if both the control and test lines appeared. A negative test was recorded if only the control line appeared. A test was considered invalid if only the treatment line appeared or if the urine did not flow up to the test band, in which case the test was repeated up to three times before recording an invalid result. The results were classified based on a test–control line comparison, where pos3 indicated a much darker test line than the control line, pos2 indicated a similar test line to the control line, pos1 indicated a fainter test line than the control line, and trace indicated a barely visible test line.

Supplementary figures

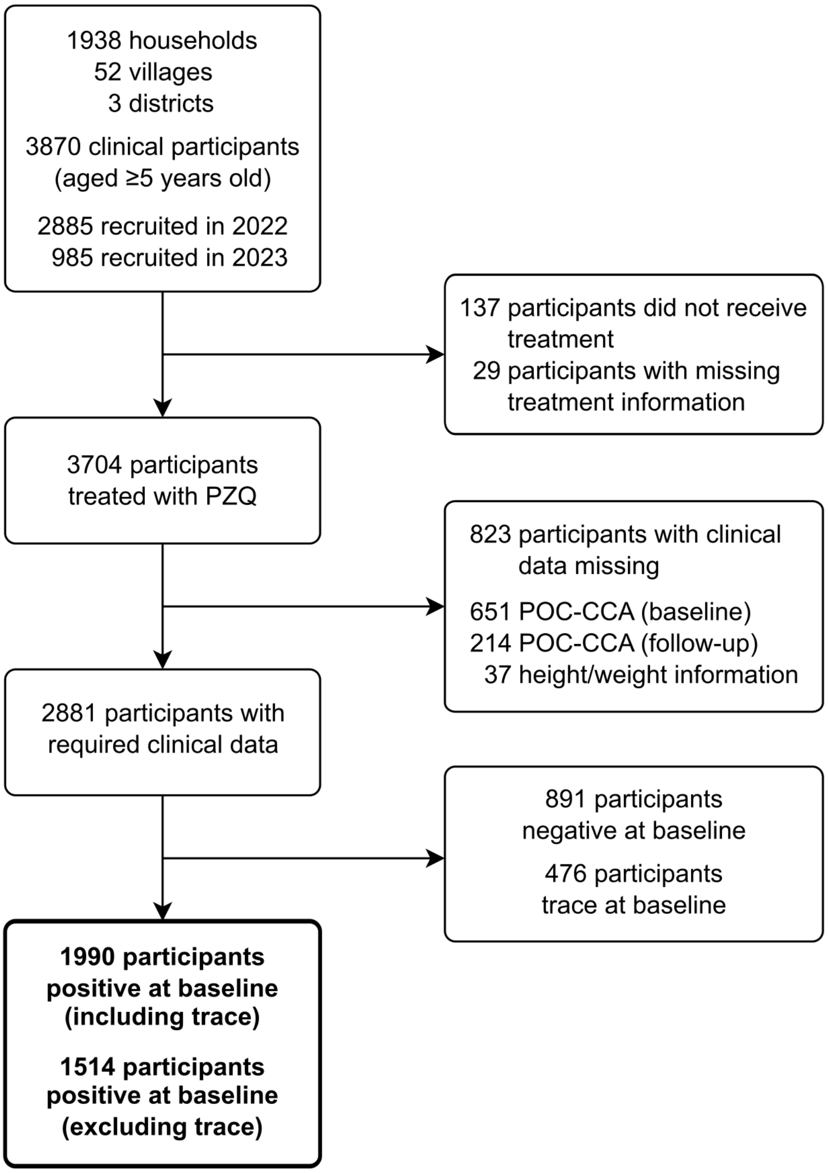

Fig. S1: **Participant flowchart (POC-CCA).** Participant numbers overlap in the breakdown of missing clinical data.

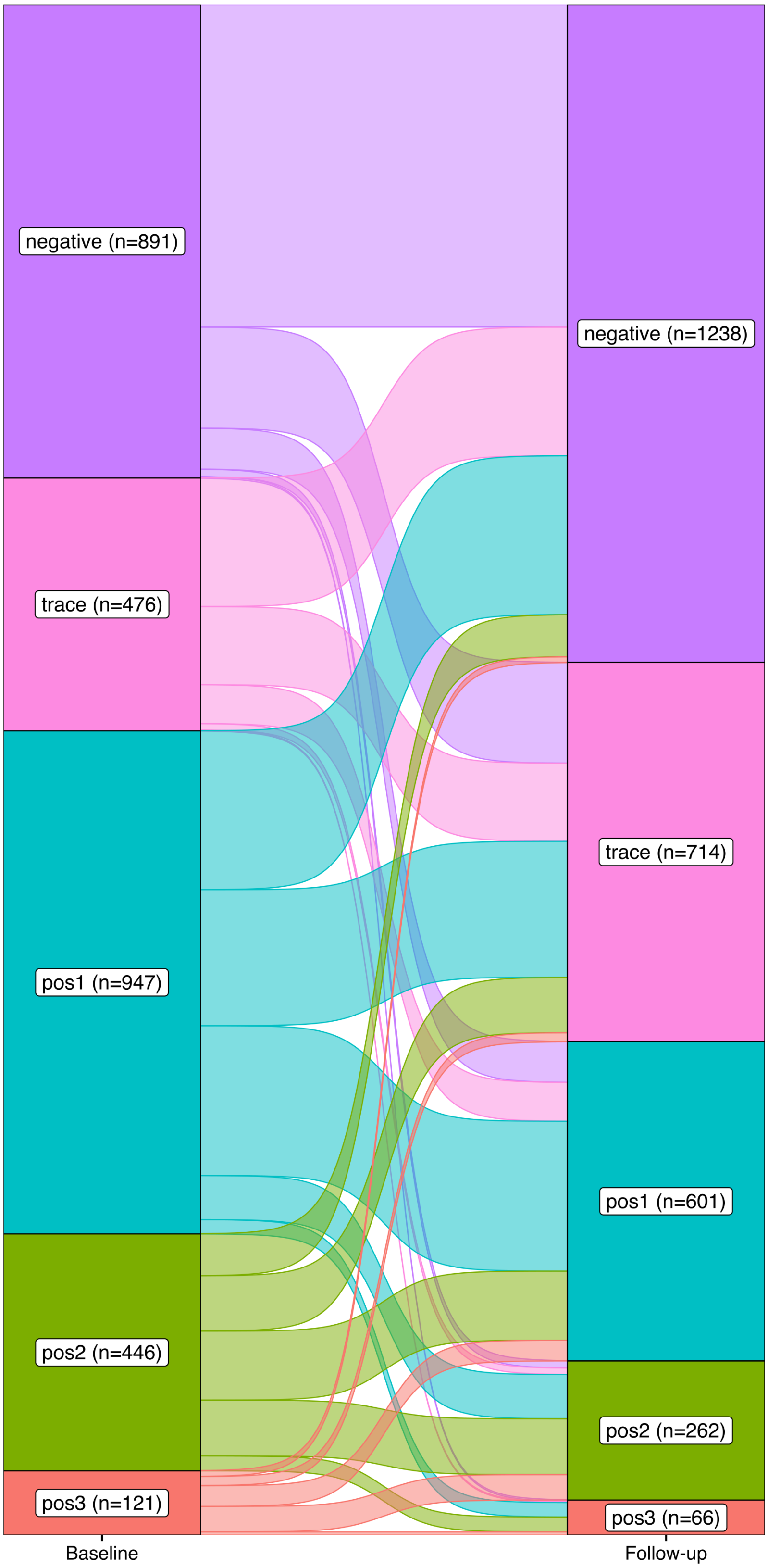

Fig. S2: **POC-CCA baseline to follow-up.** 6.3% (181/2881) of individuals were negative at baseline and positive at follow-up, when trace was considered as negative, with 9.9% (284/2881) when trace was considered as positive. 4.8% (138/2881) moved from a lower to a higher intensity when trace was considered negative, and 7.8% (225/2881) when trace was considered positive.

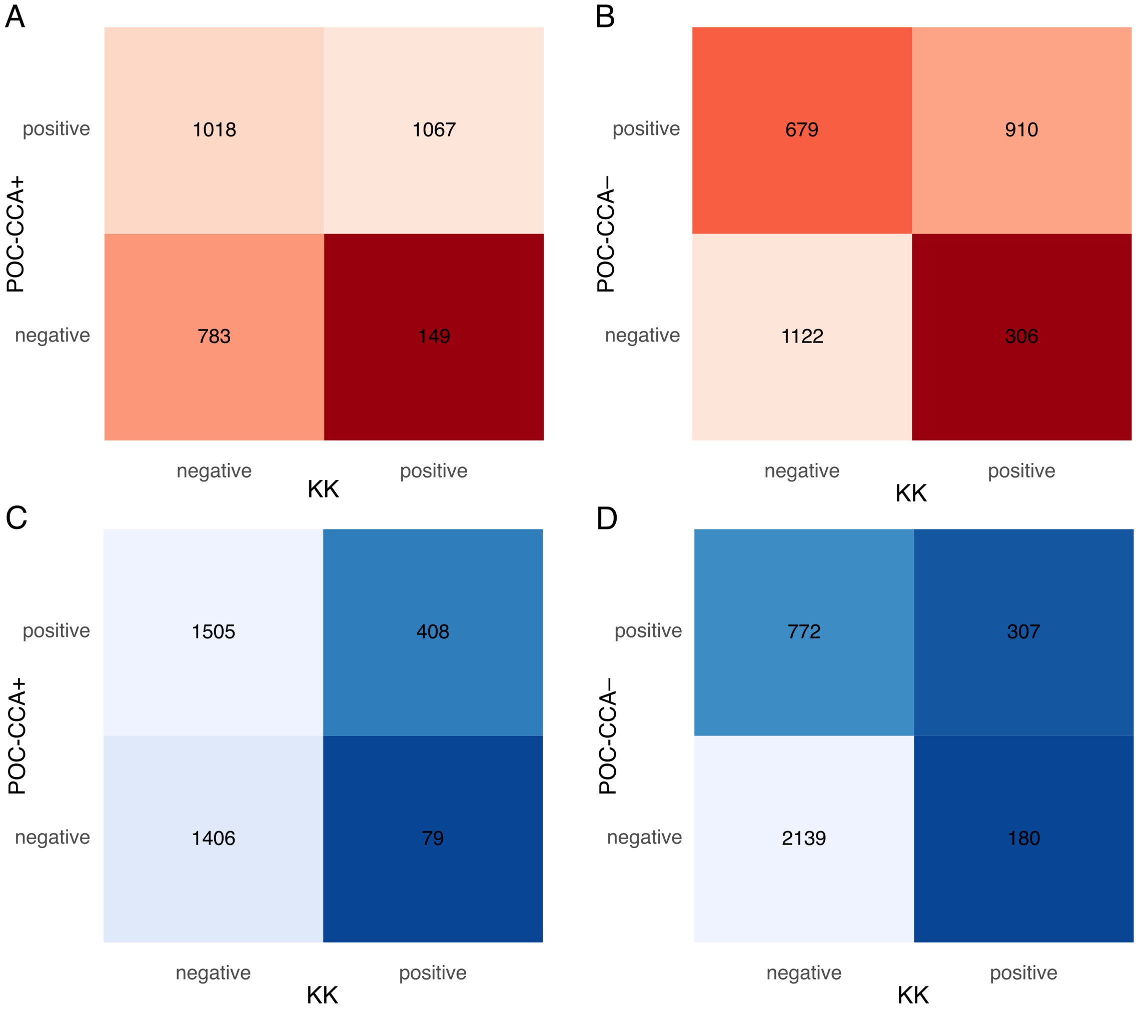

Fig. S3: **POC-CCA and Kato–Katz agreement. A, B** Show the agreement between POC-CCA (trace positive and trace negative, respectively) and KK at baseline ($n = 3017$), while **C, D** show the agreement at follow-up ($n = 3398$). Total numbers indicate the number of participants with available information out of the 3704 treated participants.

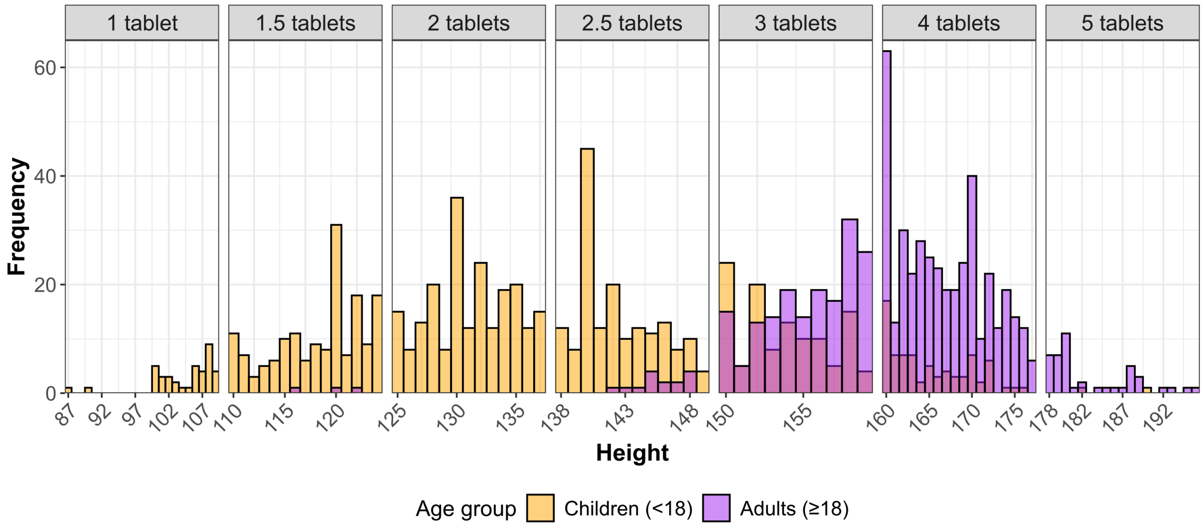

Fig. S4: **Height distribution of participants.** The distribution is visualised by age-group, with orange indicating children and purple indicating adults. Bin-widths are set at 1 cm. Each box represents a dosage category based on the WHO's dose pole, as follows: 1 tablet for $\leq$ 109 cm, 1.5 tablets for 110 to 124 cm, 2 tablets for 125 to 137 cm, 2.5 tablets for 138 to 149 cm, 3 tablets for 150 to 159 cm, 4 tablets for 160 to 177 cm, and 5 tablets for $\geq$ 178 cm.

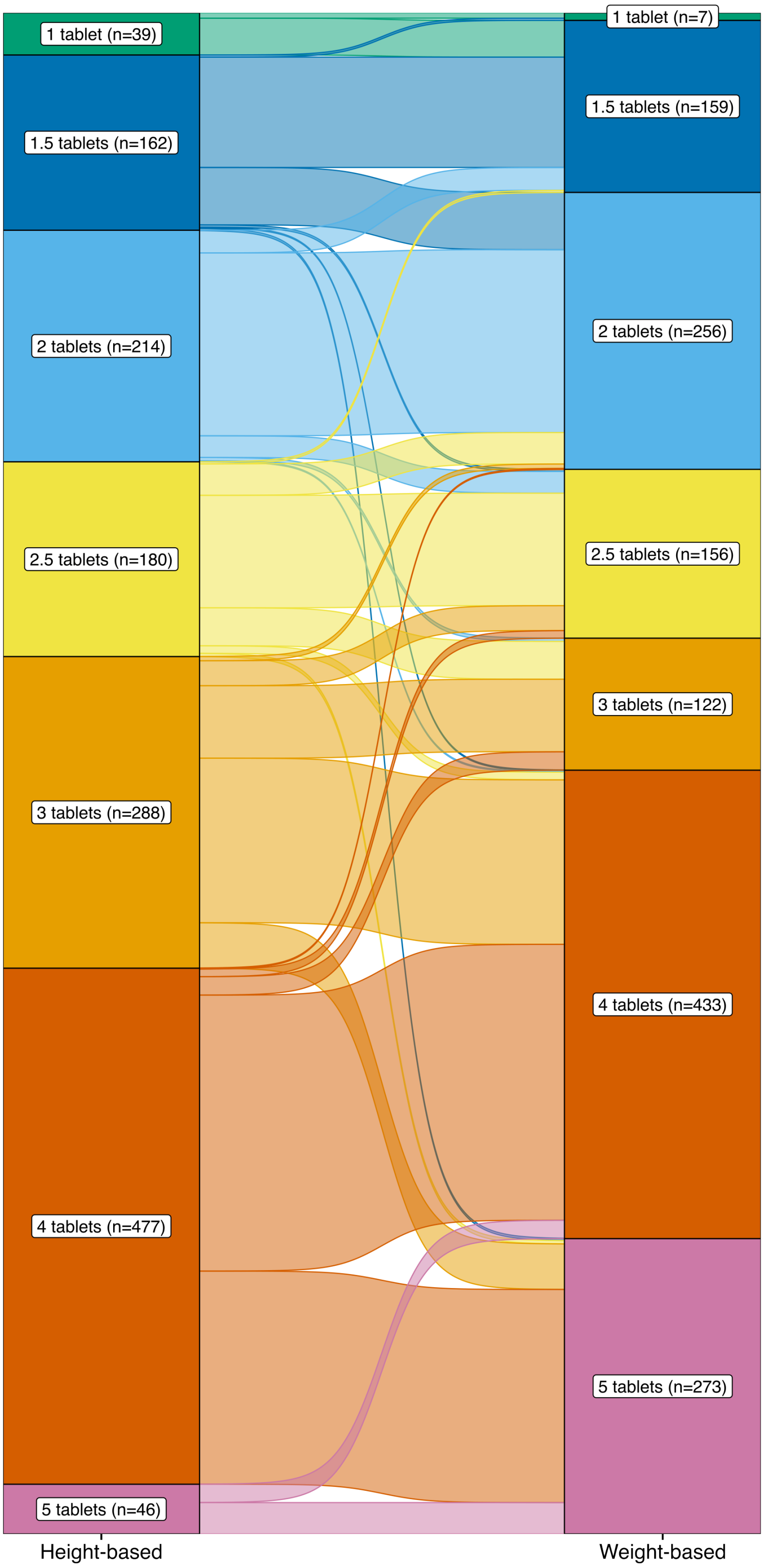

Fig. S5: **Height- vs. weight-based treatment dosage.** Each category on the left represents dosage based on the WHO's dose pole: 1 tablet for ≤ 109 cm, 1.5 tablets for 110 to 124 cm, 2 tablets for 125 to 137 cm, 2.5 tablets for 138 to 149 cm, 3 tablets for 150 to 159 cm, 4 tablets for 160 to 177 cm, and 5 tablets for ≥ 178 cm. Categories on the right are computed based on the recommended dosage of 40 mg/kg.

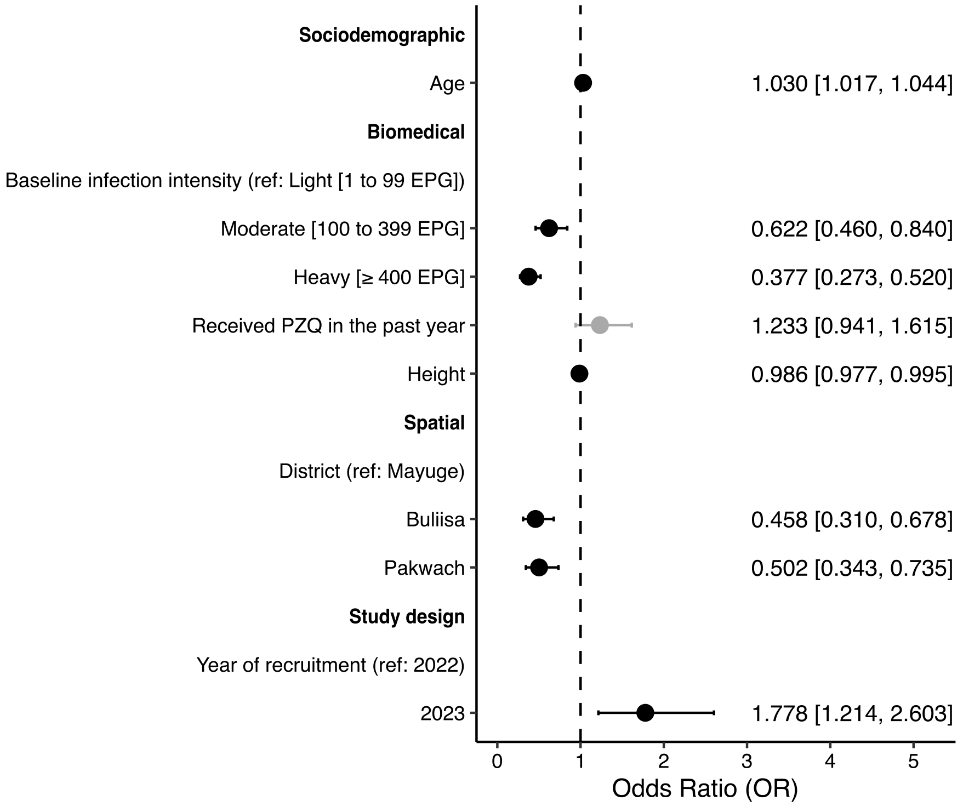

Fig. S6: **Determinants of being cured for AIC-based model.** Variable selection by step-wise backwards AIC. ORs (exponentiated coefficient estimates represented by the dots) of significant ($P < 0.05$) coefficients are shown in black, and of non-significant coefficients in grey. 95% CIs are indicated in lines. OR and CI values are also reported.

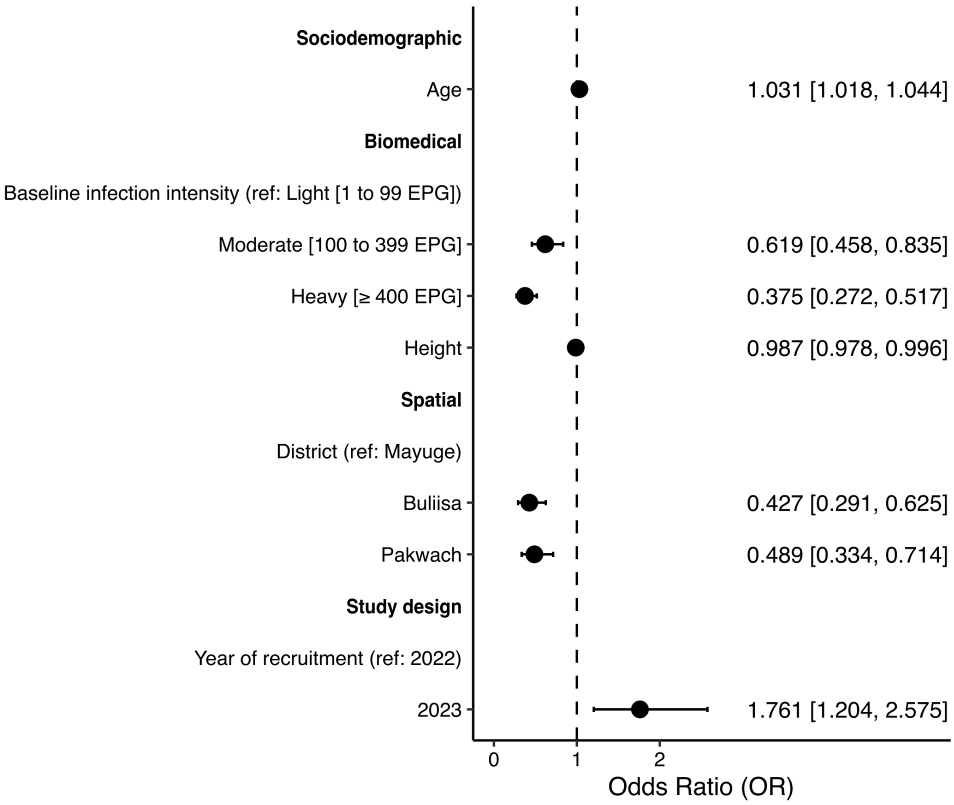

Fig. S7: **Determinants of being cured for BIC-based model.** Variable selection by step-wise backwards BIC. ORs (exponentiated coefficient estimates represented by the dots) of significant ($P < 0.05$) coefficients are shown in black, and of non-significant coefficients in grey. 95% CIs are indicated in lines. OR and CI values are also reported.

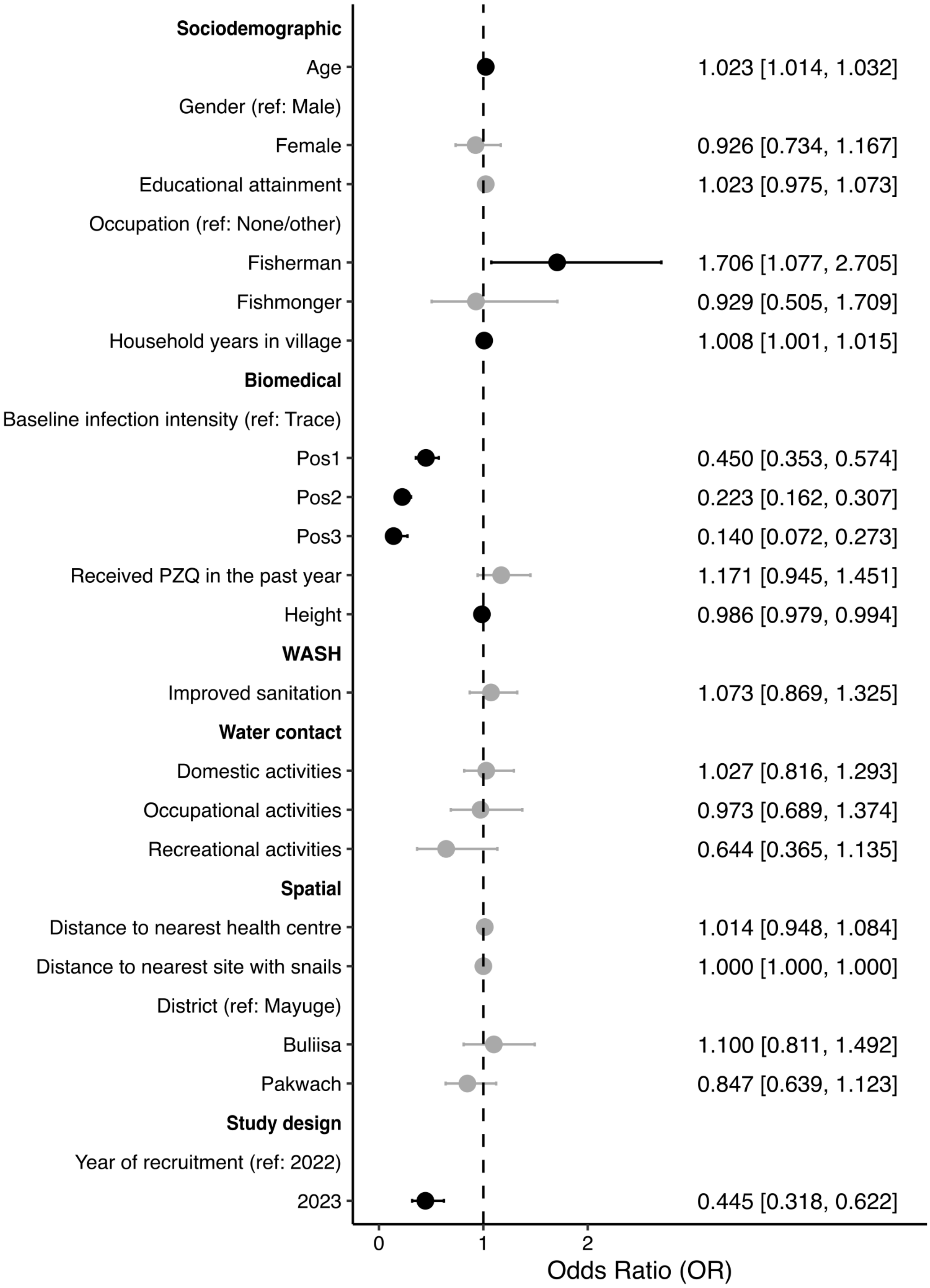

Fig. S8: **Determinants of being cured after treatment based on POC-CCA+ testing.** Trace was taken as positive. ORs (exponentiated coefficient estimates represented by the dots) of significant ($P < 0.05$) coefficients are shown in black, and of non-significant coefficients in grey. 95% CIs are indicated in lines. OR and CI values are also reported.

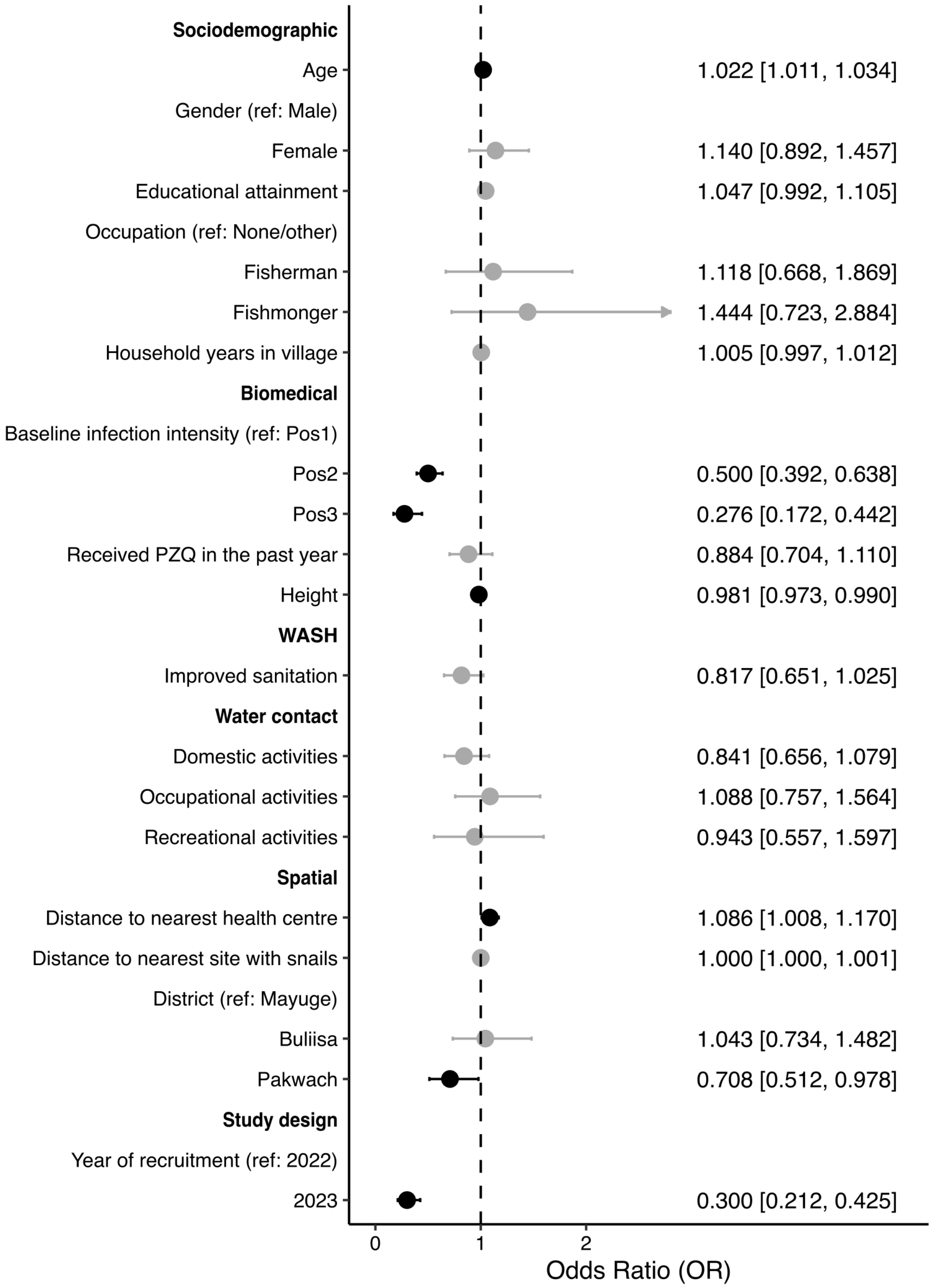

Fig. S9: **Determinants of being cured after treatment based on POC-CCA− testing.** Trace was taken as negative. ORs (exponentiated coefficient estimates represented by the dots) of significant ($P < 0.05$) coefficients are shown in black, and of non-significant coefficients in grey. 95% CIs are indicated in lines. OR and CI values are also reported.
